# Cognitive and motor outcomes in children born low birth weight: a systematic review and meta-analysis of studies from South Asia

**DOI:** 10.1186/s12887-019-1408-8

**Published:** 2019-01-29

**Authors:** Ravi Prakash Upadhyay, Gitismita Naik, Tarun Shankar Choudhary, Ranadip Chowdhury, Sunita Taneja, Nita Bhandari, Jose Carlos Martines, Rajiv Bahl, Maharaj Kishan Bhan

**Affiliations:** 1 Knowledge Integration and Translational Platform (KnIT) at Centre for Health Research and Development, Society for Applied Studies, New Delhi, India; 2 Centre for Intervention Science in Maternal and Child Health, Centre for International Health, University of Bergen, Bergen, Norway; 3 Department of Maternal, Newborn, Child and Adolescent Health, World Health Organization, Geneva, Switzerland; 4 Indian Institute of Technology (IIT), New Delhi, India; 5 Knowledge Integration and Translational Platform (KnIT), Biotechnology Industry Research Assistance Council (BIRAC), New Delhi, India

**Keywords:** Cognitive score, Motor score, Children, Adolescents, Low birth weight, South Asia

## Abstract

**Background:**

South Asia contributes substantially to global low birth weight population (i.e. those with birth weight < 2500 g). Synthesized evidence is lacking on magnitude of cognitive and motor deficits in low birth weight (LBW) children compared to those with normal birth weight (NBW) (i.e. birth weight ≥ 2500 g). The meta-analysis aimed to generate this essential evidence.

**Methods:**

Literature search was performed using PubMed and Google Scholar. Original research articles from south Asia that compared cognitive and/or motor scores among LBW and NBW individuals were included. Weighted mean differences (WMD) and pooled relative risks (RR) were calculated. All analyses were done using STATA 14 software.

**Results:**

Nineteen articles (*n* = 5999) were included in the analysis. Children < 10 years of age born LBW had lower cognitive (WMD -4.56; 95% CI: -6.38, − 2.74) and motor scores (WMD -4.16; 95% CI: -5.42, − 2.89) compared to children with NBW. Within LBW children, those with birth weight < 2000 g had much lower cognitive (WMD -7.23, 95% CI; − 9.20, − 5.26) and motor scores (WMD -6.45, 95% CI; − 9.64, − 3.27).

**Conclusions:**

In south Asia, children born LBW, especially with < 2000 g birth weight, have substantial cognitive and motor impairment compared to children with NBW. Early child development interventions should lay emphasis to children born LBW.

**Electronic supplementary material:**

The online version of this article (10.1186/s12887-019-1408-8) contains supplementary material, which is available to authorized users.

## Key notes

- Evidence is lacking from south Asian setting on magnitude of cognitive and motor deficits in low birth weight (LBW) individuals compared to those with normal birth weight (NBW).
- Our meta-analysis showed that LBW children < 10 years of age had 4.56 points lower cognitive and 4.16 points lower motor scores compared to children with NBW.
- Early child development interventions in south Asia should emphasize on children born LBW

## Introduction

Lower middle income countries (LMICs), as per the recent World Bank criteria, are those with a gross national income (GNI) per capita between USD 996 and 3895 [1]. In LMICs, around 18 million infants are born with low birth weight (LBW) (i.e. birth weight < 2500 g), of which one-fourth (26%) are in south Asia alone [2]. Infants born with low birth weight have been identified to be at an increased risk of adverse outcomes other than mortality, such as predisposition to stunting, wasting and impaired neurodevelopment outcomes [3–8]. Further, investigations based on the concept of Developmental Origins of Health and Disease (DOHaD) also link low birth weight to risk of adult onset cardiovascular, renal and metabolic disorders [9, 10].

In most of the south Asian regions, substantial thrust is still on improving survival, particularly in the neonatal period [11, 12]. In the post-neonatal period, additional inputs, either for survival or thrive, from the health system are largely lacking. Evidence on the quantum and nature of growth and development impairment in LBW infants compared to NBWs (i.e. with birth weight ≥ 2500 g) would help prioritize and aid in design of postnatal programs. The evidence from LMICs, including south Asia, is available for growth but lacking for neurodevelopment. A recent systematic review incorporating data from 137 developing countries has documented low birth weight, including prematurity and foetal growth restriction, as a leading risk factor for childhood stunting at 2 years of age [8].

Data on neurodevelopment impairment from developed countries suggest that individuals born with LBW have a higher risk of lower cognitive function, tend to score lower on academic performance measures, have higher prevalence of mental disorders, serious emotional and behavioural problems and development delay compared to term healthy counterparts [13–18]. Neurodevelopmental deficits in low birth weight infants has been linked to injury to the cerebral white matter, cystic periventricular leukomalacia, intraventricular hemorrhage, reduced total brain volume, altered cortical volume and structure, decreased total number of cells and myelination deficits [19, 20]. Brain connectivity is also impaired in such infants as evidenced by neuronal migration deficits, reduced dendritic processes, and under-efficient neural networks [19, 20]. A meta-analysis involving 15 studies (*n* = 3276) from developed settings documented lower cognitive scores in school aged children born preterm, compared to controls born at term (Weighted mean difference 10.9; 95% CI, 9.2–12.5) [21]. These findings, however, may not be entirely generalizable to south Asia, owing to the difference in settings and populations. In the developed settings, LBWs are predominantly premature whereas small for gestational age (SGA) contributes majorly to LBW in south Asia [2, 22]. Further, social factors, economic factors as well as quality of available health care could moderate the trajectory of developmental outcomes and these are different in south Asia when compared to developed settings.

Our systematic review examined the degree of developmental impairment primarily in LBW children, compared with normal birth weights, in south Asia. Additionally, a similar comparison was also done for adolescent age group. Synthesizing such comparative evidence will be helpful in strategic planning of a health program aimed at improving child development. A question in deciding about such program is whether to reach all infants equally, irrespective of their birth weights or make additional inputs on LBWs. To address this question, we attempted to elucidate how NBW children in south Asian context grow developmentally compared to NBW children from upper-middle-income settings (GNI per capita between USD 3896 to 12,055) [1]. For this, we have compared cognitive and motor scores of NBW children from south Asian settings with those from upper middle-high income settings.

## Methods

### Primary objective(s) of the systematic review

The primary objective of the systematic review was to compare cognitive and motor scores among children aged < 10 years born with normal and low birth weight in south Asian setting. It also encompassed a comparison of these outcomes between children born with a birth weight of < 2000 g and those with NBW. We further extended such comparisons until the adolescent age group (i.e. 10–19 years of age).

### Objective of the additional analysis

The objective of the additional analysis was to compare cognitive and motor scores among NBW children born in south Asia and upper middle-high income settings, following the World Bank classification [1].

### Search strategy and selection criteria

#### For the primary objective

A systematic search was performed by two authors independently (GN, TSC) using PubMed and Google Scholar. Google Scholar was used as an adjunct resource to complement PubMed as it offers advantages in terms of its potential to provide access to the gray literature, theses, abstracts, conference proceedings, preprints and institutional repositories. Any discrepancy was discussed with a third author (RPU). Search strategies used subject headings and key words with no language and time restrictions. For abstracts/articles published in non-English language, we planned to use Google translator or involve a language expert to help the team in comprehending the study findings. The search strategy is presented in Table 1. The last date of article search was 31st December 2017. The bibliographies of relevant guidelines, reviews and reports were also read to identify relevant primary reports. For studies with data missing or requiring clarification, investigators of the included studies were contacted.

**Table 1:** Search strategy used to identify articles to be included in the systematic review and meta-analysis

1.	(Neurodevelopmental OR Neurodevelopment OR Neurobehavioral OR Neurobehavioural OR Cognitive OR Intellectual OR Developmental OR Learning OR Language OR Behaviour OR Behavior OR Motor OR Motor Skill OR Movement OR Intelligence OR Psychomotor OR Psychomotor performance OR Developmental coordination OR Mental OR Memory OR Disability OR Disabilities OR Manifestations OR Disorder OR Dysfunction OR Outcome OR Retardation OR Neuropathology OR Cerebral Palsy OR Attention deficit OR Attention deficit hyperactivity disorder OR school performance OR Child development OR Infant development OR Developmental Delay OR Long term Outcome)
2.	(birthweight OR birth weight)
3.	(#1 AND #2) Filter: Customized country filter (India OR Bangladesh OR Pakistan OR Nepal OR Bhutan OR Sri Lanka OR Maldives OR Afghanistan OR south Asia)

To be included, the study had to be an original research, either cross-sectional or cohort. Studies reporting outcomes of interest by birth weight in the control arm of a randomized controlled trial were also eligible. Included studies should have been conducted in south Asian setting and have compared outcomes of interest among normal and low birth weight individuals. After initial screening of titles and abstracts, full-text publications of potential studies were reviewed. Discrepancies about inclusion of studies and interpretation of data were resolved by discussion with the other authors (RPU, RC). Data from all studies meeting the inclusion criteria were abstracted into a tabular form (RPU). Newcastle-Ottawa Quality Assessment Scale adapted for observational studies was used for quality assessment of included studies [23]. The assessment was done by two authors separately (GN and TSC). In case of any discrepancy, a third author (RPU) independently assessed the study.

#### For the additional analysis

For the additional analysis, a search strategy was developed to identify most recent reviews that either presented pooled cognitive and/or motor scores for NBW individuals or compared cognitive and/or motor scores among normal and low birth weight individuals from upper-middle-high income settings. The key search terms included: “birth weight”, “low birth weight”, “preterm”, “cognition”, “intelligence”, “motor”, “psychomotor”, “neurocognitive”, “systematic reviews”, “meta-analysis”. The search strategy was run on PubMed and Google Scholar. Last date of search was 31st December, 2017. Data on cognitive and/or motor scores from each of the studies included in the identified review(s) were tabulated.

#### Data analysis

All analyses were done using STATA 14 software. Heterogeneity of effects was assessed and quantified by the I^2^. I^2^ values > 50% were considered to represent substantial heterogeneity [24]. In cases with substantial heterogeneity, random effects model were used. Weighted mean differences (WMD) were calculated by comparing cognitive and motor scores obtained by LBWs with normal birth weight individuals. Standardized assessment tests provide raw scores on scales that are compared to same age peers for norm-referenced interpretation. Norms are often standardized to a mean of 100 and a standard deviation (SD) of 15 [25]. In studies where standardized tests were not used, the scores were converted into a standardized scale with mean of 100 and standard deviation of 15 [25, 26]. This was done to effectively pool all the studies and obtain an estimate in terms of weighted mean difference. Pooled relative risks (RR) were also calculated with normal birth weight individuals as the reference. Subgroup analysis based on birth weight i.e. birth weight < 2000 g, compared to normal birth weight, was done. All pooled estimates were reported with 95% confidence intervals.

In studies that reported an outcome at different points in time, only the outcome reported at the most recent point of assessment was considered for analysis. This was done to avoid the analyses of correlated data from repetitive and paired observations, and consequently compromising the reliability of the findings of this meta-analysis. In studies where the outcomes were reported as median (range), conversion into mean (standard deviation) was done using a reliable method [27]. Where standard deviation was not provided along with mean, it was imputed either through calculation of mean of the standard deviations from similar studies or through methods proposed by Cochrane [28, 29]. Publication bias was assessed using Begg’s test.

We did an additional analysis to compare pooled mean cognitive and motor scores among NBW children from south Asia and upper middle-high income settings. The pooled mean cognitive and motor scores for NBW individuals in these two settings were obtained separately and thereafter, compared for statistical significance of difference in means.

## Results

### Characteristics of the included studies

**Fig. 1:**
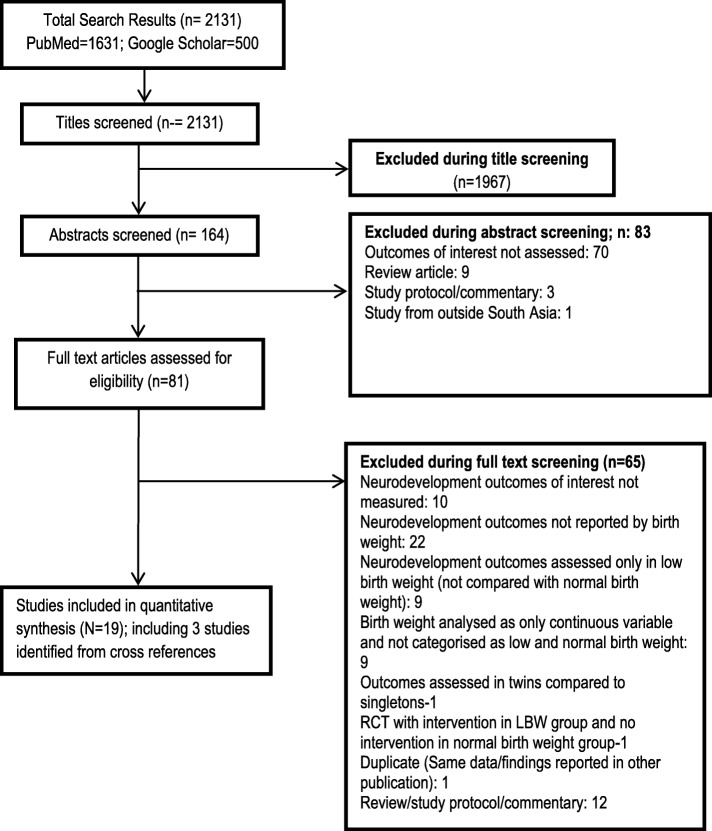
Flowchart depicting the selection process of the article for the meta-analysis

We screened 2131 titles of articles identified through electronic literature search (PubMed; *n* = 1631 and Google Scholar; *n* = 500). Out of these, 1967 were excluded based on titles and another 83 after reviewing the abstracts. We assessed 81 full text articles for eligibility and found 16 articles to be relevant for the review. Additional 3 articles were identified through cross-references of eligible studies. A total of 19 articles (with 5999 subjects; 2236 with low birth weight and 3763 with normal birth weight) were included in our final analysis [30–48]. Figure 1 shows the flowchart for article selection. All the included studies were published in English language and no additional resources were required for translation.

Out of 19 studies, 12 were conducted in India, 2 each in Pakistan, Bangladesh and Nepal and one in Sri Lanka. A total of 13 studies were conducted in children aged up to 5 years of age, three studies in children aged 6 to 9 years and 4 studies in adolescents i.e. 10–18 years of age (Table 2). One study by Tandon et al. assessed cognitive and motor outcomes in two different age groups using different set of participants i.e. involving children aged 5 to 9 years (mean, SD: 7, 1.1 years) and adolescents aged 9 to 13 years (mean, SD: 10.6; 1.2 years) (Table 2) [31]. This study was considered as two different studies for generating pooled estimates. In 11 out of 19 studies, eligible participants were enrolled into the study from hospital whereas in 8 studies, they were enrolled from community setting. A total of 13 studies involved prospective follow up of enrolled infants and children [30–33, 36, 38, 39, 41, 42, 44–46, 48]; 5 were cross-sectional studies [34, 35, 37, 43, 47] and one study involved analysis of data generated from a randomized controlled trial [40]. There were 7 studies with a quality score of ≥5. The median quality score of the included studies was 4 and scores ranged from 2 to 8.

**Table 2:** Details of the studies from south Asia included in the meta-analysis

Author (year)	Site of recruitment; Type of study	Country	Study population	Sample size	Tool(s) used	Age at assessment	Key outcome(s)	Quality score
Chaudhari (1999) [30]	Hospital; Prospective follow up	India	Infants with BW < 2000 g discharged from Neonatal special care units and full term neonates with BW > 2500 g followed up till their 6 years of age	Children with low BW- 201 Children with normal BW-71	Stanford Binet Intelligence Scale (SBIS) School report card assessment	At 6 years of age	Mean IQ score - LBW: 94.3 (13.6) - NBW: 101.38 (10.2) Proportion with abnormal IQ (score of < 85 score) - LBW: 17% - NBW: 5.6% Proportion with poor school performance (< 35% marks obtained) - LBW: 12.6% - NBW: 1.8%	6
Tandon (A)(2000) [31]	Hospital; Prospective follow up	India	Infants with BW ≤2000 g discharged from special care nursery and followed up in high risk clinics; controls were healthy term infants with BW > 2500 g followed in well baby clinics	Children with low BW:27 Children with normal BW: 28	Stanford Binet Intelligence Scale (SBIS); Raven’s Progressive Matrices; M.E Hertzig method of assessing signs of motor dysfunction	Age range of 5 to 9 years; mean age of 7.0 (SD 1.1) years	Mean cognitive score - LBW: 105.6 (13.4) - NBW: 116 (11.6) Proportion with low IQ score (<25th percentile) - LBW: 18.5% - NBW: 0.0% Proportion with signs of motor dysfunction - LBW: 37% - NBW: 10.7%	2
Tandon (B)(2000) [31]	Hospital; Prospective follow up	India	Infants with BW ≤2000 g discharged from special care nursery and followed up in high risk clinics; controls were healthy term infants with BW > 2500 g followed in well baby clinics	Children with low BW:32 Children with normal BW: 29	Stanford Binet Intelligence Scale (SBIS); Raven’s Progressive Matrices; M.E Hertzig method of assessing signs of motor dysfunction	Age range of 9 to 13 years; mean age of 10.6 (SD 1.2) years	Mean cognitive score - LBW: 99.6 (10.8) - NBW: 110.6 (7.3) Proportion with low IQ score (<25th percentile) - LBW: 25% - NBW: 3.4% Proportion with signs of motor dysfunction - LBW: 19% - NBW: 3.4%	2
Chaudhari (2004) [32]	Hospital; Prospective follow up	India	Infants with BW < 2000 g discharged from Neonatal special care units and full term neonates with BW > 2500 g and followed up till their 12 years of age	Adolescents with low BW- 180 Adolescents with normal BW-90	Weschler’s Intelligence Scale; Movement assessment battery; School report card assessment	At 12 years of age	Mean IQ score - LBW: 89.5 (16.9) - NBW: 97.2 (14.1) Proportion with abnormal IQ (score of < 85) - LBW: 37.7% - NBW: 18.8% Proportion with poor school performance (< 50% marks obtained) - LBW: 21.6% - NBW: 10.0% Mean motor impairment score - LBW: 9.8 (3) - NBW: 7.3 (2.9)	4
Juneja (2005) [33]	Hospital; Prospective follow up	India	Term infants < 2000 g and term infants with normal birth weight (> 2500 g)	Infants with BW < 2000 g-50 Infants with BW > 2500 g-30	Bayley Scales of Infant Development (BSID II)	At 18 months	Mean mental development quotient - < 2000 g: 91.5 (16.9) - > 2500 g: 102 (8.4) Mean motor development quotient - < 2000 g: 93.2 (19.7) - > 2500 g: 99.5 (10.3) Proportion with adverse mental development outcome - < 2000 g: 20% - > 2500 g: 3.3% Proportion with adverse motor development outcome - < 2000 g: 24% - > 2500 g: 3.3%	2
Taneja (2005) [34]	Community; Cross-sectional	India	Children aged 12 to 18 months enrolled in a randomized controlled trial	Children with low BW- 61 Children with normal BW-116	Bayley Scales of Infant Development (BSID II)	At 12–18 months of age Findings of assessment at baseline used	Mean mental development quotient - LBW: 102.2 (12.26) - NBW: 102.8 (11.03) Mean motor development quotient - LBW: 100.08 (13.97) - NBW: 101.06 (12.37) Proportion with abnormal mental score (score of < 85) - LBW: 4.92% - NBW: 5.17% Proportion with abnormal motor score (score of < 85) - LBW: 13.1% - NBW: 4.3%	7
Subasinghe (2006) [35]	Community; Cross-sectional	Sri Lanka	Preschool children within the age range of 36–54 months	Children with low BW: 12 Children with normal BW: 62	Early Screening Inventory for Preschoolers (ESI-P)	36 to 54 months of age	Mean cognitive score - LBW: 63.35 (14.5) - NBW: 65.32 (15.7) Mean gross motor score - LBW: 62.7 (7.4) - NBW: 68.81 (18.1)	3
Nair (2009) [36]	Hospital; Prospective follow up	India	Adolescents with known birth weight, follow up done at 13 years of age	Adolescents with low BW-183 Adolescents with normal BW-211	Raven’s coloured progressive matrices	At 13 years of age	Proportion with low IQ score (≤25th percentile) - LBW: 51.4% - NBW: 41.7%	4
Sidhu (2010) [37]	Community; Cross-sectional	India	Children aged 2 to 35 months recruited from a urban center	Children with low BW: 57 Children with normal BW: 196	Clinical Linguistic Auditory Milestone Scale (CLAMS)	2–35 months of age; mean age of 14.15 months	Mean Language Quotient (LQ)^a^ - LBW: 85.07 (16.6) - NBW: 94.66 (16.6)	3
Hoque (2012) [38]	Hospital; Prospective follow up	Bangladesh	Newborns discharged from a special care baby unit and followed till 12 months of age	Infants with low BW: 25 Infants with normal BW: 80	Bayley Scales of Infant Development (BSID II)	At 12 months of age	Mean mental score - LBW: 114.18 (12.80) - NBW: 117.11 (12.04) Mean motor score - LBW: 96.14 (25.12) - NBW: 108.41 (19.69)	4
Khan (2012) [39]	Hospital; Prospective follow up	Pakistan	Neonates discharged from neonatal intensive care unit and followed till 6 months of age	Infants with low BW: 92 Infants with normal BW: 18	Denver Development Screening Test (DDST II)	At 6 months of age	Proportion with delayed development (development quotient < 60)^b^ - LBW: 38% - NBW: 0%	4
Tofail (2012) [40]	Community; Secondary data analysis from a randomized controlled trial	Bangladesh	Live born singletons	Low BW infants- 66 Normal BW infants- 183	Bayley Scales of Infant Development (BSID II)	At 10 months of age	Mean mental index score - LBW: 99.5 (7) - NBW: 102.9 (8) Mean motor index score - LBW: 96.8 (10) - NBW: 102.7 (10)	7
Modi (2013) [41]	Hospital; Prospective follow up	India	VLBW admitted to a neonatal intensive care unit prospectively followed till 1 year of corrected age. A cohort of term, birth weight (≥2500 g) infants born during same period was enrolled for comparison.	VLBW-37 NBW-35	Developmental Assessment Scale for Indian Infants (DAS II)	At 12 months of age	Mean mental index score - VLBW: 92.9 (8.0) - NBW: 98.4 (6.1) Mean motor index score - VLBW: 90.1 (9.6) - NBW: 96.6 (5.8)	5
Chaudhari (2013) [42]	Hospital; Prospective follow up	India	Infants with BW < 2000 g discharged from Neonatal special care units and full term neonates with BW > 2500 g and followed up till their 18 years of age	Adolescents with low BW-161 Adolescents with normal BW-73	Raven’s Progressive Matrices	At 18 years of age	Mean IQ score^a^ - LBW: 39.3 (29.9) - NBW: 52.5 (29.9) Proportion with low IQ score (<25th percentile) - LBW: 24.2% - NBW: 12.7% Poor school performance (failed at least in one standard in school) - LBW: 25.5% - NBW: 5.5%	4
Avan (2014) [43]	Community; Cross-sectional	Pakistan	Low birth weight and normal birth weight infants	Low BW infants-86 Normal BW infants-566	Bayley Scales of Infant Development (BSID II)	Within 3 years of age	Mean psychomotor development index score - In low BW: 94.13 (18.13) - In normal BW: 98.47 (15.84)	6
Nair (2014) [44]	Hospital; Prospective follow up	India	Infants discharged from Neonatal special care units and followed up with 12 months of age	Infants with low BW- 170 Infants with normal BW-429	Developmental Assessment Scale for Indian Infants (DAS II)	At 12 months of age	Mean mental index score^a^ - LBW: 107.83 (11.04) - NBW: 110.51 (8.38) Mean motor index score^a^ - LBW: 99.72 (14.28) - NBW: 104.17 (10.86)	5
Christian (2014) [45]	Community; Prospective follow up	Nepal	Children aged 7 to 9 years who were part of an earlier nutrition supplementation trial	Children with low BW-764 Children with normal BW-1163	UNIT for general intelligence; Finger tapping test for fine motor	At 7 to 9 years of age (mean age of 8.4 years)	Mean Intelligence score (UNIT) - LBW: 47.6 (9.4) - NBW: 51.6 (10.1) Mean fine motor score - LBW: 35.8 (5.4) - NBW: 36.9 (5.1) Mean motor impairment score - LBW: 9.98 (6.73) - NBW: 7.62 (5.59)	8
Chattopadhyay (2015) [46]	Hospital; Prospective follow up	India	Newborns discharged from SNCU	Children with low BW- 206 Children with normal BW-181	TDSC DDST II Visual and hearing assessment	Under 3 years of age	Proportion with developmental delay - LBW: 38.8% - NBW: 20.9%	4
Singh (2017) [47]	Community; Cross-sectional	India	Children under 2 years of age from an urbanized village	Children with low BW- 43 Children with normal BW-153	Ages and Stages questionnaire, 3rd Edition	Under 2 years of age	Proportion with development delay - LBW: 16.3% - NBW: 2.0%	4
Kvestad (2017) [48]	Community; Prospective follow up	Nepal	Infants aged 2–12 months enrolled through a cross-sectional survey and followed up till 5 years of age	Children with low BW: 124 Children with normal BW: 193	Ages and Stages Questionnaire, 3rd edition	At 5 years of age	Mean cognitive score - LBW: 52.68 (6.9) - NBW: 51.61 (9.1) Mean motor score - LBW: 53.44 (6.1) - NBW: 53.37 (7.5)	4

*BW* birth weight, *LBW* low birth weight, *VLBW* very low birth weight, *NBW* normal birth weight, *IQ* intelligence quotient

^a^SD calculated using imputation method (http://handbook-5-1.cochrane.org/chapter_7/7_7_3_3_obtaining_standard_deviations_from_standard_errors.htm)

^b^Developmental delay was assessed based on the cumulative score of developmental quotient (DQ) for each of the four domains (i.e. gross motor, language, fine motor and personal/social skills) and dividing by 4. A score of < 60 were labelled as “developmentally delayed”. DQ was calculated as, (developmental age/corrected chronological age)*100. Developmental age was established depending on the degree of achievement in each domain; UNIT-Universal Nonverbal Intelligence Test; CO = − cohort; RCT- randomized controlled trial; TDSC- Trivandrum Developmental Screening Chart; DDST- Denver Developmental Screening tool

### Findings of the cognitive score

**Fig. 2:**
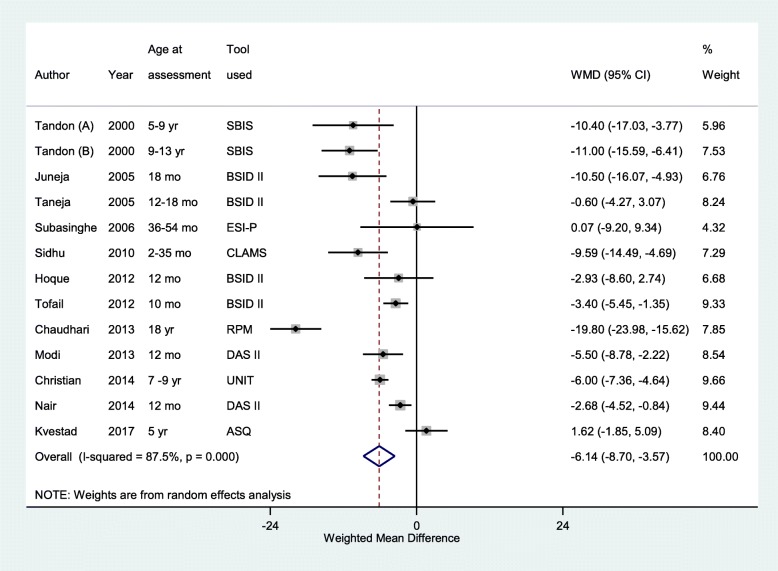
Overall pooled weighted mean difference (WMD) of cognitive scores from infancy till adolescence in individuals born low birth weight compared to those born with normal birth weight

**Fig. 3:**
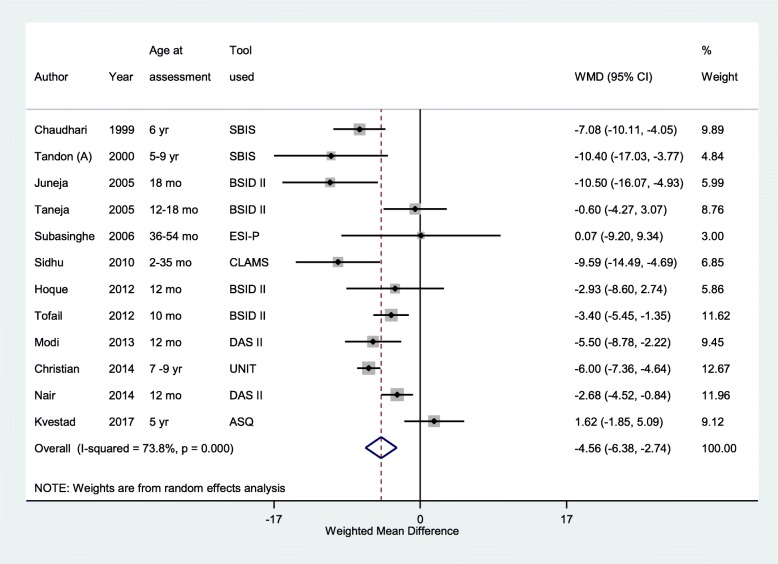
Pooled weighted mean difference (WMD) in cognitive scores in children aged < 10 years born with low birth weight, compared to their counterparts with normal birth weight

The overall pooled weighted mean difference (WMD) in cognitive scores from infancy till adolescence in low birth weights, compared to NBW participants was − 6.14 (95% CI; − 8.70, − 3.57) (*n* = 4203, I^2^ = 87.5%) (Fig. 2). Children under 10 years of age born with low birth weight had around 5 points lower cognitive scores compared to NBW children (Weighted mean difference (WMD) -4.56; 95% CI; − 6.38, − 2.74) (*n* = 4180; I^2^ = 73.8%) (Fig. 3). The difference among low and normal birth weights in cognitive scores was even higher, though with wider confidence intervals, in the adolescent age group (WMD -15.45; 95% CI; − 24.08, − 6.83) (*n* = 295, I^2^ = 87.1%).

The proportion with low cognitive score, defined as IQ score of less than 25th percentile or a mental quotient of < 85, was 14% (95% CI; 6–22%) and 5% (95% CI; 2–8%) in LBW and NBW children aged < 10 years respectively (Data not shown). The risk of low cognitive score in children under 10 years was around 2.5 times higher in LBWs compared to those born with NBW (RR 2.69; 95% CI, 1.34–5.39) (*n* = 584, I^2^ = 12.7%) (Table 3). This risk was 1.28 times higher in adolescents (aged 10–18 years) born LBW compared to those born NBW (RR 1.28; 95% CI, 1.02–1.61) (*n* = 687, I^2^ = 46.8%).

**Table 3:** Risk of adverse neuro-developmental outcomes in children < 10 years of age born with low birth weight compared to those born with normal birth weight

Outcomes	No. of studies	No. of subjects	Age range	Pooled RR (95% CI)	I ^2^ Statistic
Low cognitive score^a^	4	584	< 10 years	2.69 (1.34, 5.39)	12.7%
Motor impairment^b^	3	312	< 10 years	3.32 (1.56, 7.06)	0.0%
Developmental delay^c^	3	693	≤3 years	1.97 (1.41, 2.73)	69.3%

^a^Defined as mental quotient of < 85 or IQ score ≤ 25th percentile

^b^defined as either presence of signs of motor dysfunction on clinical examination or motor quotient of < 85

^c^defined as developmental quotient of < 60 on developmental screening tools and/or presence of visual/hearing/speech difficulties

### Findings of the motor score

**Fig. 4:**
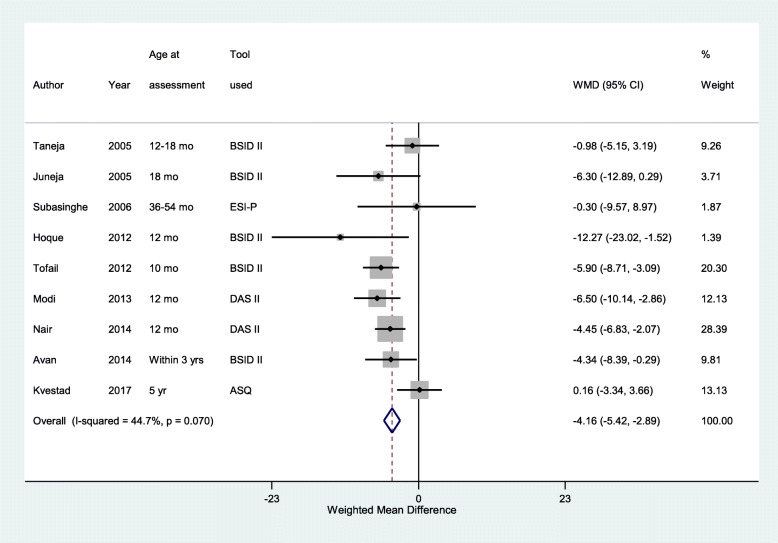
Pooled weighted mean difference (WMD) in motor scores in children under-five years of age born low birth weight, compared to those with normal birth weight

Children, under-five years of age, born LBW had 4 points lower motor scores compared to children with NBW (WMD -4.16, 95% CI; − 5.42, − 2.89) (*n* = 2325, I^2^ = 44.7%) (Fig. 4). Among children < 10 years of age, 23% (95% CI; 10–35%) of LBW children had motor impairment (defined as either presence of signs of motor dysfunction on clinical examination or motor quotient of < 85) as opposed to 5% of normal birth weight children (95% CI; 1–8%). The risk of motor impairment in children born LBW was around 3 times higher compared to those born NBW (RR 3.32; 95% CI, 1.56–7.06) (*n* = 312, I^2^ = 0.0%) (Table 3).

### Findings of cognitive and motor scores in a sub-group of LBW (< 2000 g)

**Fig. 5:**
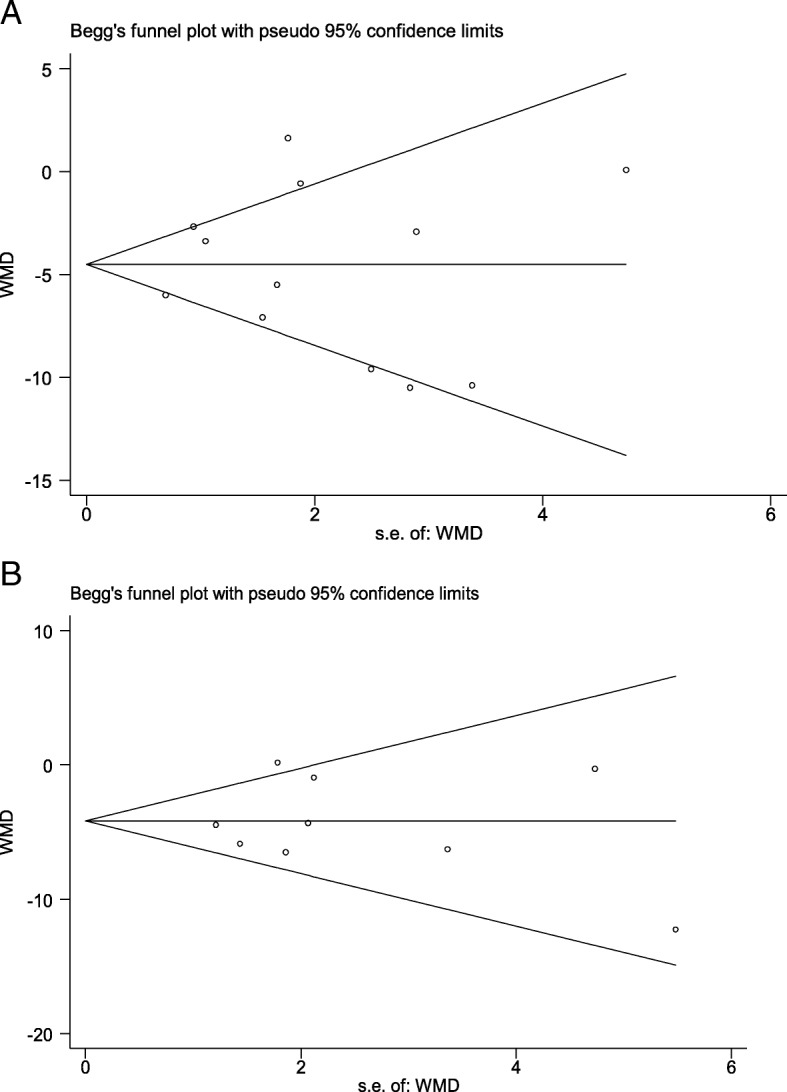
Begg’s graph to examine evidence of publication bias for primary outcomes. **a** WMD for cognitive score in children aged < 10 yrs.; **b** WMD for motor score in children aged < 10 yrs.

Within LBW children under 10 years of age, those with birth weight < 2000 g had much lower cognitive and motor scores when compared to children with normal birth weight (Table 4). Such children had around 7 points lower cognitive score (WMD -7.23, 95% CI; − 9.20, − 5.26) (*n* = 479, I^2^ = 8.7%) compared to their counterparts with NBW (Table 4). The risk of low cognitive performance was nearly 4 times higher (RR 3.59; 95% CI; 1.55, 8.32) (*n* = 407; I^2^ = 0.0%). In terms of motor performance, such children had around 6.5 points lower motor score compared to their NBW counterparts (WMD -6.45, 95% CI; − 9.64, − 3.27) (*n* = 152; I^2^ = 0.0%). There was around 4 times higher risk of low motor performance in children born with birth weight of < 2000 g (RR 3.72, 95% CI; 1.32, 10.54) compared to those with a weight of ≥2500 g at birth (*n* = 135; I^2^ = 0.0%) (Table 4). Additional findings from the studies included in the review have been presented in Additional file 1: Table S1. Begg’s plot did not suggest publication bias for the primary outcomes of interest (*P* value of 0.837 and 0.917 for WMD cognitive and WMD motor scores respectively) (Fig. 5).

**Table 4:** Risk of adverse neuro-developmental outcomes in children < 10 years of age born with birth weight < 2000 g compared to those born with normal birth weight (≥2500 g)

Outcomes	No. of studies	No. of subjects	Age range	Effect size (95% CI)	I ^2^ Statistic
Cognitive score	4	479	< 10 years	WMD −7.23 (−9.20; −5.26)	8.7%
	3	407	< 10 years	RR 3.59 (1.55; 8.32)^a^	0.0%
Motor score	2	152	< 10 years	WMD −6.45 (−9.64; −3.27)	0.0%
	2	135	< 10 years	RR 3.72 (1.32; 10.54)^b^	0.0%

^a^Represents the risk of having “low cognitive performance” defined as mental quotient of < 85 or IQ score ≤ 25th percentile

^b^Denotes the risk of having “low motor performance” defined as either presence of signs of motor dysfunction on clinical examination or motor quotient of < 85

### Findings of the additional analysis

For the additional analysis, the search strategy identified three systematic reviews for cognition and one for motor performance. The search strategy used to identify systematic reviews from upper middle-high income settings resulted in a total of 690 articles of which 53 were duplicates. Another 606 articles were rejected based on title screening. Full texts of 31 reviews were read and of them 4 were included for the additional analysis [49–52]. There were four studies from South Asia [35, 42, 45, 48] wherein the reported scores were converted into standardized scores with mean of 100 and SD of 15 in order to make them comparable to those reported in studies from upper middle-high income settings.

Mean cognitive scores for NBW children aged < 10 years in upper middle-high income countries was 105.37 (95% CI; 103.54, 107.20) and for south Asia it was 104.13 (95% CI; 100.94, 107.31) with a *P*-value for difference in means of 0.482 (Additional file 1: Table S2, Figure S1 and Figure S2). The overall pooled mean cognitive scores in NBW individuals from infancy till adolescence for upper middle-high income countries and south Asia were 104.56 (95% CI; 103.34, 105.78) and 105.03 (95% CI; 101.96, 108.10) respectively (*P*-value for difference in means 0.799) (Additional file 1: Figures S3 and S4).

Mean motor scores for NBW children < 10 years of age from upper middle-high income countries and south Asia were 106.89 (95% CI; 101.39, 112.40) and 101.75 (95% CI; 99.45, 104.05) respectively (*P*-value for difference in means = 0.092) (Additional file 1: Table S3, Figures S5 and S6).

## Discussion

The current meta-analysis was done to primarily assess the magnitude of cognitive and motor impairment that children born with low birth weight experience, compared to their normal birth weight counterparts in a south Asian setting. We observed that LBW children had 5 point lower cognitive scores and 4 point lower motor scores compared to children with normal birth weight. The deficit in scores was even greater (around 7 points) in those with birth weight of < 2000 g. The risk of cognitive and motor deficits in LBWs seemed to persist throughout the transition from early childhood to adolescence. In the additional analysis, we found cognitive and motor scores of NBW children from south Asian settings to be similar to those in upper middle-high income settings.

There is substantial difference in quantum of deficits that LBW individuals experience compared to normal birth weights, in upper middle-high income and south Asian settings. Recently published systematic reviews from upper middle-high income settings document LBW individuals to have around 8–12 points lower cognitive scores, as opposed to 5 points lower scores found by us in south Asia [49–51]. Similarly, children and adolescents who were LBW had around 13 points lower motor scores in upper middle-high income settings whereas we found a 4 to 6 points lower motor scores [52]. These observed differences could be due to the difference in the nature of low birth weights in these two settings. While small for gestational age (SGA) forms a significant proportion of LBW in south Asia, prematurity contributes to a major proportion in high income/developed settings [2, 22].

The magnitude of cognitive deficit is often related to subsequent income. According to the World Bank 2006 report, studies from United States, Pakistan, Kenya and Tanzania reported 8–12, 6.5, 8 and 5% decreases in wages, respectively, for a 0.5 SD decline in cognitive score [53]. In the present metaanalysis, we found an overall pooled 0.40 SD (around 6 points; 1 SD = 15 points on standardized developmental assessment scales) decrease in cognitive scores from infancy to adolescence in LBWs and therefore, could possibly expect a similar reduction in adult wages. The amount of economic potential lost is a serious concern and therefore demands early recognition and action.

South Asia is home to a large proportion of the World’s LBW infants [2]. Until now, much of the focus has been on ensuring survival of these vulnerable subsets, particularly in the neonatal period, as mortality rates are high in the first few months of life [6, 54]. Those who survive this critical period are often cared for similarly to infants born with NBW and no additional efforts are made to improve their growth and development. It seems imperative to consider how to design programs wherein the needs of such infants can be met. One key policy question in the south Asian context, considering the constraints in resources, is to decide whether early child development interventions should be for all children irrespective of their birth weight or primarily for those with low birth weight. The findings of the study underscore the need to target LBW children. Further, as shown in the additional analysis, NBW children in south Asia appear to reach similar cognitive and motor scores as observed in upper middle-high income settings. A similar finding was reported by a recent multicentre study from India, Argentina, Turkey and South Africa, where most developmental milestones in normal birth weight healthy children in early childhood were attained at similar ages across these four diverse settings [55]. While we believe that early child development (ECD) intervention program(s) should cater to the growth and developmental needs of all children yet if we are to reduce the inequity, mechanisms for additional care focussing on LBW infants, particularly those with birth weight of under 2000 g, should be ensured.

To the best of our knowledge, this is the first systematic review which documents the magnitude of deficits in cognitive and motor performance among individuals born from south Asia with LBW compared to those with NBW. The current review suffers from paucity of good quality studies from south Asia; only a third of the included studies had a score of ≥5 out of 10 (reflecting acceptable quality). Another limitation of the review is that because of the limited data on cognitive and motor performance disaggregated by prematurity and SGA, we could not compare their separate neurodevelopment outcomes with term-appropriate for gestational age (term-AGA) infants. Further, the small number of studies reporting outcomes in adolescence led to wide confidence intervals of estimates. Most of the studies in the review were from India with lesser representation from other countries of south Asia. However, the social, cultural, economic and health care milieu in most of the south Asian countries has strong similarities and therefore, the generalizability of the findings will probably remain unaffected. We used Google Scholar to complement the findings of the search on PubMed. However, we acknowledge the limitations of Google Scholar such as lack of reliable advanced search functions, lack of controlled vocabulary (similar to MeSH terms in PubMed) and inadequate understanding of the exact scope of its coverage. The included studies varied in the nature of study design. Almost all the studies were observational in nature; however, pooling of studies with cohort and cross-sectional design might lead to some loss of reliability of the findings. In studies with prospective follow up, we would expect the birth weight to have been reliably measured while birth weight data may be prone to recall bias and consequent misclassification of birth weight categories in cross-sectional studies (especially when data is not recorded from hospital birth records). In studies where standardized test was not used, the scores were converted into a standardized scale with mean of 100 and standard deviation of 15. The advantage of this conversion is that scores from different tests can be meaningfully interpreted and compared. However, there is a limitation that such conversions assume a normal distribution but in case this assumption is not met, the scores cannot be interpreted as a standard proportion of the distribution from which they were calculated.

## Conclusions

This metaanalysis from south Asian setting reveals significant deficits in cognitive and motor scores in children and adolescents born with low birth weight, compared to those born with normal birth weight. We also observed a dose effect relationship wherein among the LBW children; those with birth weight of less than 2000 g had much lower cognitive and motor scores. While NBW infants from south Asia appear to develop similar to their counterparts from upper middle-high income settings, the high degree of deficits among LBWs underscore the need for prioritizing the delivery of child development interventions to these children.

## Additional file

Additional file 1: **Table S1.** Summarized additional findings in the studies included in the meta analysis. **Table S2.** Mean cognitive scores in normal birth weight children and adolescents from upper middle-high income settings and south Asia. **Table S3.** Mean motor scores in normal birth weight children from upper middle-high income and south Asian setting. **Figure S1.** Pooled mean cognitive scores in children < 10 years of age born with normal birth weight (≥2500 g) from upper middle-high income settings. **Figure S2.** Pooled mean cognitive scores in children < 10 years of age born with normal birth weight (≥2500 g) from south Asian setting. **Figure S3.** Pooled mean cognitive scores from infancy till adolescence in individuals born with normal birth weight (≥2500 g) from upper middle-high income settings. **Figure S4.** Pooled mean cognitive scores from infancy till adolescence in individuals born with normal birth weight (≥2500 g) from south Asian setting. **Figure S5.** Pooled mean motor scores in children < 10 years of age born with normal birth weight from upper middle-high income settings. **Figure S6.** Pooled mean motor scores in children < 10 years of age born with normal birth weight from south Asian setting. (DOC 221 kb)

## Abbreviations

AGA: Appropriate for gestational age
CENTRAL: Central register for controlled trials
ECD: Early child development
LBW: Low birth weight
LMIC: Low middle income countries
NBW: Normal birth weight
RR: Relative risk
SGA: Small for gestational age
WMD: Weighted mean difference
