# Supplementary material for: Cognitive and motor outcomes in children born low birth weight: a systematic review and meta-analysis of studies from South Asia
Source: BMC Pediatr. 2019 Jan 29;19:35. doi: 10.1186/s12887-019-1408-8 (PMC6350290; doi:10.1186/s12887-019-1408-8)
Supplement: Supplementary file 1 — Table S1. Summarized additional findings in the studies included in the meta analysis. Table S2. Mean cognitive scores in normal birth weight children and adolescents from upper middle-high income settings and south Asia. Table S3. Mean motor scores in normal birth weight children from upper middle-high income and south Asian setting. Figure S1. Pooled mean cognitive scores in children < 10 years of age born with normal birth weight (≥2500 g) from upper middle-high income settings. Figure S2. Pooled mean cognitive scores in children < 10 years of age born with normal birth weight (≥2500 g) from south Asian setting. Figure S3. Pooled mean cognitive scores from infancy till adolescence in individuals born with normal birth weight (≥2500 g) from upper middle-high income settings. Figure S4. Pooled mean cognitive scores from infancy till adolescence in individuals born with normal birth weight (≥2500 g) from south Asian setting. Figure S5. Pooled mean motor scores in children < 10 years of age born with normal birth weight from upper middle-high income settings. Figure S6. Pooled mean motor scores in children < 10 years of age born with normal birth weight from south Asian setting. (DOC 221 kb) [file 12887_2019_1408_MOESM1_ESM.doc]

**Supplementary material to the manuscript titled “Cognitive and motor outcomes in children born low birth weight: a systematic review and meta-analysis of studies from south Asia”**

**Table S1 . Summarized additional findings in the studies included in the meta-**analysis

| **Study** | **Additional findings** |
| --- | --- |
| **Chaudhari, 1999**  LBW = 201  NBW = 71 | Intelligence Quotient: Assessed by Stanford Binet Scales of Intelligence  The score of Intelligence Quotient was significantly lower in Full term SGA (96.02±15.4), Preterm AGA (95.85±14.6), Preterm SGA (92.22±11.6) compared to full term normal birth weight children (101.38±10.2).  Visual Motor Function: Assessed by Bender Gestalt (Higher score is worse)  LBW children (10.57:t 3.5) had significantly higher score compared to normal birth weight (8.9: t 3.5) children.  Emotional state: Assessed by Koppitz test  No significant difference of abnormal findings of Koppitz test among LBW (17.5%) compared to normal birth weight children (13.1%)  Occupational Therapy Assessment by *Ayres and Bobath*  Higher proportion of LBW children (16.9%) required for assistance for language assessment compared to normal birth weight children (5.6%).  16 LBW children had poor preschool skills compared to none in the normal birth weight children. |
| **Tandon, 2000**  **Group 1**  LBW = 27  NBW = 28  **Group 2**  LBW = 32  NBW = 29 | **Group 1: Age in years, Mean (SD) 7 (1.1)**  Intelligence Quotient: Assessed by Raven’s Progressive matrices  No significant difference in IQ score among LBW children (mean ±SD: 106.6±10.8) and normal birth weight children (mean ±SD:116.6±10.8).    Academic Achievement: Assessed by WRAT R.  The score of reading was significantly lower in LBW children (mean ±SD:108±14.7) compared to normal birth weight children (mean ±SD:117.3±14.7).  The score of spelling was significantly lower in LBW children (mean ±SD:108.8±14.9) compared to normal birth weight children (mean ±SD:117.9±11.5).  The score of Arithmetic was significantly lower in LBW children (mean ±SD:113.4±14.9) compared to normal birth weight children (mean ±SD:125.7±10.4).  Visual Motor Function: Assessed by Bender Gestalt  Higher proportion of LBW children (33%) are dysfunction compared to normal birth weight children (7.1%)  Social Maturity: Assessed by Vineland Scale Vineland Scale  No difference in Social Quotient score among LBW children (mean ±SD: 107.3±13.3) and normal birth weight children (mean ±SD:117.7±9.3).  **Group 2: Age in years, Mean (SD) 10.6 (1.2)**  Intelligence Quotient: assessed by Raven’s Progressive matrices  The score of IQ among LBW children (mean ±SD: 99.4±11.7) was significantly lower normal birth weight children (mean ±SD:111.8±9.1) assessed by Raven’s Progressive matrices  Academic Achievement: Assessed by WRAT R.  The score of reading was significantly lower in LBW children (mean ±SD:96±12.2) compared to normal birth weight children (mean ±SD:111.1±10.3).  The score of spelling was significantly lower in LBW children (mean ±SD:95.7±12.0) compared to normal birth weight children (mean ±SD:110.5±9.9).  The score of Arithmetic was significantly lower in LBW children (100.2±13.8) compared to normal birth weight children (mean ±SD:115.2±13.3).  Visual Motor Function: Assessed by Bender Gestalt  Higher proportion of LBW children (49%) are dysfunction compared to normal birth weight children (21%)  Social Maturity: Assessed by Vineland Scale  The Social Quotient score was significantly lower among LBW children (mean ±SD: 99.4±11.3) compared to normal birth weight children (mean ±SD:111.7±8.5). |
| **Chaudhari, 2004**  LBW = 180  NBW = 90 | Visual Motor Function: Assessed by Bender Gestalt  Higher proportion of LBW children (46.6%) with visuo-motor age less than 9 years as compared to normal birth weight children (30%).  23.3% LBW children with age appropriate visuo-motor perception compared to 47.8% in normal birth weight children.  Specific learning disability assessed by Wide Range Achievement Test  Higher proportion of LBW (18.3%), VLBW (25%), and preterm (22.7%) children had poor writing skills compared to normal birth weight children (6%)  The score of mathematics skill is significantly lower (mean ±SD: 82.7±16.9) compared to normal birth weight children (mean ±SD: 87.8±15.8)  Motor Function: Assessed by Movement Assessment Battery  The score was significantly higher in LBW children [median (IQR): 9.8(0-18)] compared to normal birth weight children [median (IQR): 7.3 (0-17.5)]    Emotional or Behavioral problem: Assessed by Draw-a-person Screening Procedure for  Emotional Disturbance (DAP-SPED)  Higher proportion of LBW children (42.9%) showed emotional instability compared normal birth weight children (32.7%). |
| **Juneja, 2005** | 2 LBW children had visual Impairment (cortical blindness = 1; abnormal VEP=1); (LBW; n = 50); No case among NBW children (NBW; n = 30) |
| **Nair, 2009**  LBW = 200  NBW = 224 | Intelligence Quotient: Assessed by Raven’s Progressive matrices  Statistically significant difference in intelligence scores assessed by Raven’s Progressive Color Matrices at 13 years was present only in male adolescents.  46.2% LBW children (Male) performed well compared to 59.7% normal birth weight children (Male).  Among Preterm, 92.2% Appropriate for gestational Age (AGA) adolescents had good performance compared 7.8% small for gestational age in adolescent. |
| **Hoque, 2012**  LBW = 25  NBW = 80 | Behavior Rating: Assessed by BSID II  None of the behavior rating (Activity, Approach, Co-operation, Emotional tone, Vocalization) was significantly different among LBW babies compared to Normal birth weight babies. |
| **Tofail, 2012**  LBW = 66  NBW = 183 | Behavior Rating: Assessed by Bayley test using a scale modified and designed by Wolke  Normal birth weight children had improved behavior rating compared to LBW children [Adjusted beta-coefficient (95%CI): 0.5(0.1–0.8)]  *Adjusted for: Age, sex, asset index, stimulation received at home, fish oil capsule intake, gestational age, maternal BMI, birth weight group.* |
| **Khan, 2012**  LBW = 57  VLBW = 29  ELBW = 6  ALL LBW = 92  NBW = 18 | **Developmental status: Assessed by Denver Development Screening Test II**  **Gestation**  Proportion of infants with developmental delay in different types of premature babies;  Extremely premature (<28 wk.): 100% (n = 4)  Severe preterm: 70% (n =33)  Moderate preterm: 15% (n = 39)  Late preterm: 6% (n = 34  **Birth Weight**  Proportion of infants with developmental delay in different birth weight categories:  NBW: 0% (n = 18)  LBW: 9% (n = 57)  VLBW: 83% (n = 29)  ELBW: 100% (n = 6)  **Fetal Growth restriction**  Proportion of infants with developmental delay in different types fetal growth  SGA: 52% (n= 14)  AGA: 33.3% (n=21) |
| **Chaudhari, 2013**  LBW = 161  NBW = 73 | Aptitude: Assessed by Differential Aptitude Test  LBW children were poor in speed compared to normal birth weight children.  Mechanical reasoning: Preterm SGA had significantly low scores compared to normal birth weight children. Full term SGA had significantly low scores in space relations & speed compared to normal birth weight children. |
| **Christian, 2014**  LBW = 764  NBW = 1163  SGA = 1052  AGA = 848  Term = 1923  Preterm = 408 | General Intelligence: Assessed by Universal Nonverbal Intelligence Test (UNIT)  Executive functioning: Assessed by Stroop test and backward digit span  Gross and fine motor abilities: Assessed by The Movement Assessment Battery for Children (MABC) and Finger tapping test.  **Fetal Growth restriction (SGA compared to No SGA)**  SGA was associated with significant lower score [Adjusted Difference (SE): −2.04(0.39)] on the test of intelligence, higher failure (6%) on the Numbers Stroop test, lower score [Adjusted Difference (SE): -0.16(0.04)] backward digit span, higher MABC score [Adjusted Difference (SE): 0.98(0.25)] and lower Finger tapping score [Adjusted Difference (SE): -0.66(0.22)]  **Birth Weight (LBW compared Normal birth weight)**  LBW was associated with significant lower score [Adjusted Difference (SE): −1.63(0.40)] on the test of intelligence, higher failure (6%) on the Numbers Stroop test, lower score [Adjusted Difference (SE): -0.16(0.04)] backward digit span and higher MABC score [Adjusted Difference (SE): 1.29(0.31)].  LBW children had lower but insignificant difference finger tapping score [Adjusted Difference (SE): -0.25(0.23)] compared to normal birth weight children.  **Gestation (Preterm compared to Term)**  Preterm children had lower score [Adjusted Difference (SE): −0.07 (0.46)] on the test of intelligence, no difference on the Numbers Stroop test, lower score [Adjusted Difference (SE): -0.06(0.05)] backward digit span, higher MABC score [Adjusted Difference (SE): 0.11(0.31)] and higher Finger tapping score [Adjusted Difference (SE): 0.02(0.26)].  All differences are insignificant.  *Adjusted child age, sex, ever started school, intake of dark green leafy vegetables, intake of citrus, maternal literacy, maternal Raven’s score, parity, asset score, history of diarrhea/dysentery, household salt iodine level.* |
| **Singh,2017**  LBW = 43  NBW = 153 | Developmental Delay: Assessed by ASQ-3  **Gestation**  Proportion of Preterm with developmental delay 10% and Term children: 4.5% |

LBW- Low birth weight; VLBW- very low birth weight; ELBW; extremely low birth weight; SGA- small for gestational age; AGA- appropriate for gestational age; IQ- intelligence quotient; VEP- visual evoked potential; ASQ-3- ages and stages questionnaire,3^rd^ edition; BSID II- Bayley Scales of Infant Development, 2^nd^ edition; WRAT R; Wide Range Achievement Test Revised; IQR- inter-quartile range; SD- standard deviation; SE- standard error

**Table S2: Mean cognitive scores in normal birth weight children and adolescents from upper middle-high income settings and s**outh Asia

| **Author (Year)** | **Setting** | **Age at assessment** | **Sample size** | | **Scale used** | **Mean (SD) score obtained** |
| --- | --- | --- | --- | --- | --- | --- |
| **UPPER MIDDLE-HIGH INCOME SETTING** | | | | | | |
| Yi KH et al (2016) | South Korea | 12 yrs | 46 | WISC-III | | 109.52 (12.54) |
| Serenius et al (2016) | Sweden | 6.5 yrs | 367 | WISC-IV | | 100.3 (11.7) |
| Molloy et al (2014) | Australia | 18 yrs | 159 | WASI | | 106.46 (13.72) |
| Ritter et al (2014) | Switzerland | 10 yrs | 36 | WISC (HAWIK-IV) | | 109.28 (7.77) |
| Guarini et al (2014) (A) | Italy | 6 yrs | 60 | K-BIT | | 96.7 (11.4) |
| Guarini et al (2014) (B) | Italy | 8 yrs | 26 | K-BIT | | 106.5 (9.4) |
| McNicholas et al (2013) | Ireland | 11 yrs | 48 | WISC-IV | | 101.3 (11.7) |
| Cheong et al(2013) | Australia | 18 yrs | 132 | WASI | | 107.6 (12.8) |
| Hutchinson et al (2013) | Australia | 8 yrs | 173 | WISC-IV | | 105.6 (12.4) |
| Lundequist et al (2013) | Sweden | 5 yrs | 117 | WPPSI-R | | 102.3 (11.0) |
| Aarnoudse-Moens et al (2013) | Netherlands | 8 yrs | 230 | WISC-III | | 105 (13.4) |
| Munck et al (2012) | Finland | 5 yrs | 168 | WPPSI-R | | 111.7 (14.5) |
| Potharst et al (2011) | Netherland | 5 yrs | 95 | WPPSI | | 103 (11) |
| Ni et al (2011) | China | 6 yrs | 22 | WISC-IV | | 103.9 (11.1) |
| Mullen et al (2011) | USA | 16 yrs | 41 | WISC-III | | 104.56 (16.31) |
| Lohaugen et al (2010) | Norway | 19 yrs | 81 | WAIS-III | | 101 (12) |
| Hallin et al (2010) | Sweden | 18 yrs | 54 | WAIS-III | | 105.70 (12.5) |
| Soria-Pastor (2009) | Spain | 9 yrs | 22 | WISC-IV | | 121.9 (15.3) |
| Aarnoudse-Moens et al (2009) | Netherland | 6 yrs | 50 | WPPSI-R | | 109 (19.2) |
| Woodward et al (2009) | New Zealand | 4 yrs | 107 | WPPSI-R | | 104.7 (13.45) |
| Kontis et al (2009) | UK | 19 yrs | 45 | WASI | | 104.48 (13.04) |
| Ehrenstein et al (2009) | Denmark | 19 yrs | 16932 | Boerge Prien | | 100.32 (15) |
| Mu et al (2008) | China (Taiwan) | 8 yrs | 59 | WISC-III | | 111.05 (14.81) |
| Gaddlin et al (2008) | Sweden | 15 yrs | 57 | WISC-III | | 97.1 (13.3) |
|  |  |  | 28 |  |  | 85.7 (14.7) |
| Allin et al (2008) | UK | 19 yrs | 44 | WASI | | 105.3 (12.8) |
| Saavalainen et al (2007) | Finland | 9 yrs | 31 | WISC-R | | 100.3 (10.6) |
| Narberhaus et al (2007) | Spain | 14 yrs | 53 | WISC-III or WAIS-III | | 113.6 (11.5) |
| Schothorst et al (2007) | Netherland | 16 yrs | 20 | WISC-R | | 109.80 (12.15) |
| Hoff et al (2006) | Denmark | 5 yrs | 76 | WPPSI-R | | 107.3 (11.4) |
| Breslau et al (2006) | USA | 17 yrs | 308 | WAIS-III | | 98.44 (13.86) |
| Martinez-Cruz et al (2006) | Mexico | 6 yrs | 41 | Stanford Binet | | 106.8 (11.7) |
| Caldu et al (2006) | Spain | 13 yrs | 25 | WISC-R or WAIS-III | | 113.3 (12.2) |
| Samuelsson et al (2006) | Sweden | 15 yrs | 52 | WISC-III | | 97.10 (13.20) |
| Nosarti et al (2006) | UK | 16 yrs | 59 | WISC-III | | 100.83 (13.34) |
| Hack et al (2005) | USA | 8 yrs | 176 | KABC | | 99.8 (15) |
| Lefebvre et al (2005) | France | 18 yrs | 44 | WAIS | | 108 (14) |
| Marlow et al (2005) | UK | 6 yrs | 160 | K-ABC | | 105.7 (11.8) |
| Caravale et al (2005) | Italy | 3-4 yrs | 30 | Stanford Binet | | 121 (10.6) |
| Kilbride et al (2004) | USA | 4 yrs | 25 | Stanford Binet | | 95 (11) |
| Taylor et al (2004) | USA | 16 yrs | 52 | WISC-III or WAIS-III | | 97.9 (16.2) |
| Anderson et al (2003) | Australia | 8 yrs | 220 | WISC-III | | 104.9 (14.1) |
| Short et al (2003) | USA | 8 yrs | 99 | WISC-III | | 101.9 (15) |
| Cooke et al (2003) | UK | 7 yrs | 198 | WISC-III | | 100.5 (13.7) |
| Grunau et al (2002) | Canada | 9 yrs | 30 | WISC-R | | 117.3 (13) |
| Magill-Evans et al (2002) | Canada | 10 yrs | 23 | WISC-III | | 101.5 (11.9) |
| Breslau (2001) | USA | 11 yrs | 163 | WISC-R | | 112.8 (14.3) |
|  |  |  | 143 |  | | 94.1 (13.6) |
| Rickards et al (2001) | Australia | 14 yrs | 41 | WISC-III | | 105 (13.3) |
| Nadeau et al (2001) | Canada | 5 yrs | 44 | MIQS | | 112.8 (16.2) |
| Taylor et al (2000) | USA | 11 yrs | 49 | KABC | | 106.24 (14.3) |
| Saigal et al (2000) | Canada | 14 yrs | 124 | WISC-R | | 102 (13) |
| Hughes et al (1999) | USA | 9 yrs | 188 | WISC-R | | 99.79 (16.51) |
| Stjernqvist et al (1999) | Sweden | 10 yrs | 61 | WISC-III-R | | 106.5 (15.0) |
| Wolke and Meyer (1999) | Germany | 6 yrs | 264 | KABC | | 99.7 (11.2) |
| **SOUTH ASIAN SETTING** | | | | | | |
| Kvestad et al (2017) | Nepal | 5 yrs | 193 | | ASQ-3 | 102.4 (15.3)* |
| Nair et al (2014) | India | 12 mo | 429 | | DAS-II | 110.51 (8.38) |
| Christian (2014) | Nepal | 7-9 yrs | 1163 | | UNIT | 102.4 (15.2)* |
| Modi et al (2013) | India | 12 mo | 35 | | DAS-II | 98.4 (6.1) |
| Chaudhari et al (2013) | India | 18 yrs | 73 | | RPM | 106.75 (15.7)* |
| Tofail et al (2012) | Bangladesh | 10 mo | 183 | | BSID-II | 102.9 (8) |
| Hoque et al (2012) | Bangladesh | 12 mo | 80 | | BSID-II | 117.11 (12.04) |
| Sidhu (2010) | India | 14.15 mo** | 196 | | CLAMS | 94.66 (16.6) |
| Subasinghe et al (2006) | Sri Lanka | 36-54 mo | 62 | | ESI-P | 99.9 (15)* |
| Taneja et al (2005) | India | 12-18 mo | 116 | | BSID-II | 102.8 (11.03) |
| Juneja et al (2005) | India | 18 mo | 30 | | BSID-II | 102 (8.4) |
| Chaudhari et al (2004) | India | 12 yrs | 90 | | WISC | 97.2 (14.1) |
| Tandon et al (2000) (A) | India | 7 yrs | 28 | | Stanford Binet | 116 (11.6) |
| Tandon et al (2000) (B) | India | 10 yrs | 29 | | Stanford Binet | 110.6 (7.3) |
| Chaudhari et al (1999) | India | 6 yrs | 71 | | Stanford Binet | 101.38 (10.2) |

*Scores reported in the study have been transformed into a scale with mean 100 and SD 15 ; **mean age at assessment; WISC-III: Wechsler Intelligence Scale for Children, Third Edition; WISC-R: Wechsler Intelligence Scale for Children, Revised; WISC-IV: Wechsler Intelligence Scale for Children, Fourth Edition; WPPSI: Wechsler Preschool and Primary Scales of Intelligence Test; WPPSI-R: Wechsler Preschool and Primary scale of intelligence, Revised; WASI: Wechsler Abbreviated Scales of Intelligence; WAIS-III: Wechsler Adult Intelligence Scale; KABC, Kaufman Assessment Battery for Children; K-Bit: Kaufman Brief Intelligence Test, Italian version; MIQS: McCarthy IQ Scale; ASQ-3: Ages and Stages Questionnaire, third edition; DAS-II: Developmental Assessment Scale for Indian Infants, second edition; UNIT: Universal Nonverbal Intelligence Test; RPM: Raven’s Progressive Matrices; BSID-II: Bayley Scales of Infant Development, second edition; CLAMS: Clinical Linguistic Auditory Milestone Scale; ESI-P: Early Screening Inventory for Preschoolers

**Fig S1: Pooled mean cognitive scores in children <10 years of age born with normal birth weight (≥2500 g) from upper middle-high income settings (N=3078)**

**Fig S2: Pooled mean cognitive scores in children <10 years of age born with normal birth weight (≥2500 g) from south Asian setting (N=2586)**

**Fig S3: Pooled mean cognitive scores from infancy till adolescence in individuals born with normal birth weight (≥2500 g) from upper middle-high income settings (N=21998)**

**Fig S4: Pooled mean cognitive scores from infancy till adolescence in individuals born with normal birth weight (≥2500 g) from south Asian setting (N=2617)**

**Table S3: Mean motor scores in normal birth weight children from upper middle-high income and s**outh Asian setting

| **Author (Year)** | **Setting** | **Age at assessment** | **Sample size** | **Scale used** | **Mean (SD) score obtained** |
| --- | --- | --- | --- | --- | --- |
| **UPPER MIDDLE-HIGH INCOME SETTING** | | | | | |
| Short et al (2003) | USA | 8 yrs | 99 | BOTMP | 111.7 (16.17)* |
| Holsti et al (2002) | Canada | 9 yrs | 18 | BOTMP | 111.55 (16.15)* |
| Dewey et al (1999) | Canada | 8-9 yrs | 30 | BOTMP | 102.1 (15.21)* |
| Singer et al (1997) | USA | 3 yrs | 123 | BSID-II | 103 (15) |
| **SOUTH ASIAN SETTING** | | | | | |
| Kvestad et al (2017) | Nepal | 5 yrs | 193 | ASQ-3 | 105.1 (15.5)* |
| Avan (2014) | Pakistan | Under 3 yrs | 566 | BSID-II | 98.47 (15.84) |
| Nair et al (2014) | India | 12 mo | 429 | DAS-II | 104.17 (10.86) |
| Modi et al (2013) | India | 12 mo | 35 | DAS-II | 96.6 (5.8) |
| Tofail et al (2012) | Bangladesh | 10 mo | 183 | BSID-II | 102.7 (10) |
| Hoque et al (2012) | Bangladesh | 12 mo | 80 | BSID-II | 108.41 (19.69) |
| Subasinghe et al (2006) | Sri Lanka | 36-54 mo | 12 | ESI-P | 100 (15)* |
| Taneja et al (2005) | India | 12-18 mo | 116 | BSID-II | 101.06 (12.37) |
| Juneja et al (2005) | India | 18 mo | 30 | BSID-II | 99.5 (10.3) |

BOTMP:Bruininks-Oseretsky Test of Motor Proficiency; ASQ-3: Ages and Stages Questionnaire, third edition; BSID-II: Bayley Scales of Infant Development, second edition; DAS-II: Developmental Assessment Scale for Indian Infants, second edition; ESI-P: Early Screening Inventory for Preschoolers; *Scores reported in the study have been transformed into a scale with mean 100 and SD 15

**Fig S5: Pooled mean motor scores in children <10 years of age born with normal birth weight from upper middle-high income settings (N=270)**

**Fig S6. Pooled mean motor scores in children <10 years of age born with normal birth weight from south Asian setting (N=1644)**
